# Long-Term Iron Deficiency and Dietary Iron Excess Exacerbate Acute Dextran Sodium Sulphate-Induced Colitis and Are Associated with Significant Dysbiosis

**DOI:** 10.3390/ijms22073646

**Published:** 2021-03-31

**Authors:** Awad Mahalhal, Michael D. Burkitt, Carrie A. Duckworth, Georgina L. Hold, Barry J. Campbell, David Mark Pritchard, Chris S. Probert

**Affiliations:** 1 Department of Molecular and Cellular Cancer Medicine, Institute of Systems, Molecular and Integrated Biology, University of Liverpool, Liverpool L69 3GE, UK; awad.mahalhal@liverpool.ac.uk (A.M.); dmpritch@liverpool.ac.uk (D.M.P.); 2 Department of Anatomy and Histology, Faculty of Medicine, Benghazi University, Benghazi, Libya; 3 Division of Diabetes Endocrinology and Gastroenterology, Faculty of Biology Medicine and Health, University of Manchester, Manchester M13 9PL, UK; michael.burkitt@manchester.ac.uk; 4 Molecular Physiology and Cell Signalling, Institute of Systems, Molecular and Integrative Biology, University of Liverpool, Liverpool L69 3GE, UK; carried@liverpool.ac.uk; 5 Microbiome Research Centre, St George & Sutherland Clinical School, Clinical Sciences (Pitney) Building, University of New South Wales Sydney, Kogarah, NSW 2217, Australia; georgina.hold@unsw.edu.au; 6 Department of Infection Biology & Microbiomes, Institute of Infection, Veterinary and Ecological Sciences, University of Liverpool, Liverpool L69 3GE, UK; bjcampbl@liverpool.ac.uk

**Keywords:** iron, diet, intestinal inflammation, inflammatory bowel disease, fecal microbiota

## Abstract

Background: Oral iron supplementation causes gastrointestinal side effects. Short-term alterations in dietary iron exacerbate inflammation and alter the gut microbiota, in murine models of colitis. Patients typically take supplements for months. We investigated the impact of long-term changes in dietary iron on colitis and the microbiome in mice. Methods: We fed mice chow containing differing levels of iron, reflecting deficient (100 ppm), normal (200 ppm), and supplemented (400 ppm) intake for up to 9 weeks, both in absence and presence of dextran sodium sulphate (DSS)-induced chronic colitis. We also induced acute colitis in mice taking these diets for 8 weeks. Impact was assessed (i) clinically and histologically, and (ii) by sequencing the V4 region of *16S* rRNA. Results: In mice with long-term changes, the iron-deficient diet was associated with greater weight loss and histological inflammation in the acute colitis model. Chronic colitis was not influenced by altering dietary iron however there was a change in the microbiome in DSS-treated mice consuming 100 ppm and 400 ppm iron diets, and control mice consuming the 400 ppm iron diet. Proteobacteria levels increased significantly, and Bacteroidetes levels decreased, in the 400 ppm iron DSS group at day-63 compared to baseline. Conclusions: Long-term dietary iron alterations affect gut microbiota signatures but do not exacerbate chronic colitis, however acute colitis is exacerbated by such dietary changes. More work is needed to understand the impact of iron supplementation on IBD. The change in the microbiome, in patients with colitis, may arise from the increased luminal iron and not simply from colitis.

## 1. Introduction

Inflammatory bowel disease (IBD) is a debilitating, relapsing-remitting long-term condition of the gastrointestinal tract that affects around 240,000 people in the UK [1,2,3]. Approximately one-third of patients develop iron deficiency anemia because of intestinal bleeding and/or malabsorption [4,5,6,7]. Iron deficiency is associated with a poor quality of life, fatigue, breathlessness, chest pain, and restless legs, as well as poor concentration and subfertility [8]. Iron absorption and metabolism is tightly regulated as iron is toxic [9]. The absorption of iron is influenced by serum ferritin. Ferritin ought to be low in iron deficiency, however it is an acute phase protein and its level is increased by inflammation such as active IBD, furthermore, hepcidin expression is also increased: these changes impair iron absorption exacerbating iron deficiency and complicating its diagnosis [10].

Iron deficiency may be treated effectively by intravenous or oral iron replacement therapy [11]. Different routes of administration have different side effect profiles [12]. In general, 10–15% of oral iron is absorbed by the small bowel [13] depending on requirement for iron. Unabsorbed iron leads to gastrointestinal side effects by generating free radicals in the colon [14] and stimulating the growth of some bacteria, and host infection by key enteric pathogens, including *Escherichia coli* pathovars [15,16,17].

Others have reported that, in mice, a low iron diet alters the abundance of the Prevotellaceae and Porphyromonadaceae families [18]. Thus, it appears that both low and high levels of luminal iron may influence the microbiome.

Unabsorbed oral iron supplements and gastrointestinal bleeding may result in an increase in luminal iron which may exacerbate IBD [19] and lead to increased proliferation and virulence of some bacteria [20,21,22]. Abnormal microbiota composition and decreased complexity of the gut microbiota are common features of patients with IBD, including both Crohn’s disease or ulcerative colitis [23]. Bacterial dysbiosis within the intestine is also associated with relapse of IBD [24,25] but it is not clear whether relapsing inflammation leads to dysbiosis by modulating luminal iron [26].

We hypothesized that iron supplementation (and or bleeding) in IBD patients could change the composition of the gut microbiota and potentially influence the natural history of IBD. To investigate this, we assessed the effects of long-term alterations of dietary iron on intestinal microbiota in murine models of colitis to eliminate any confounding factors based on background genetics that would be inevitable in a human population.

## 2. Results

### 2.1. Chronic Colitis in C57BL/6 Mice Is Not Influenced by Altering Dietary Iron

**Figure 1:**
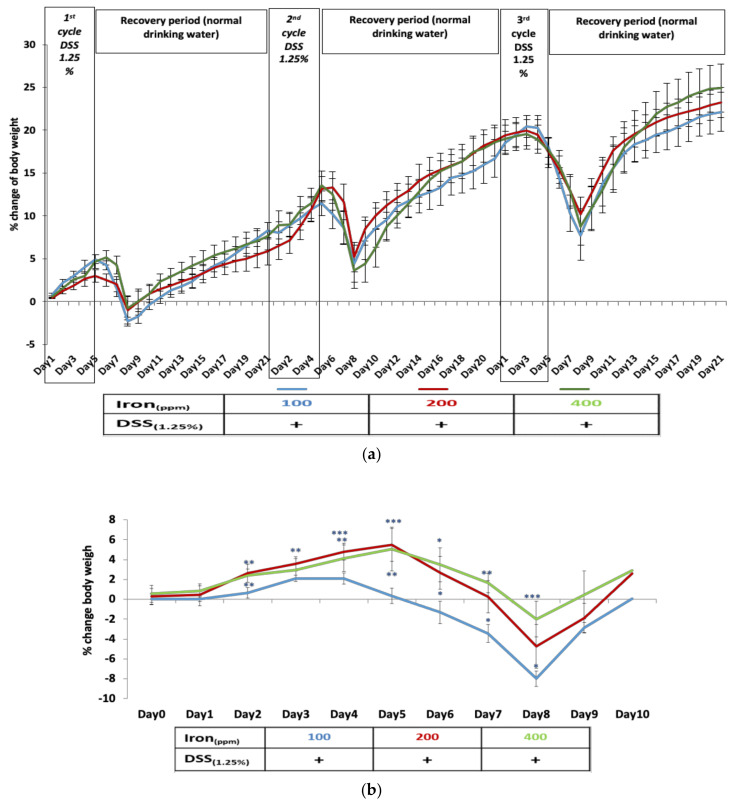
Dextran sulfate sodium (DSS) colitis – induced body weight changes in mice consuming diets of differing dietary iron levels. (**a**) Percentage weight loss observed in mice with chronic colitis induced by three cycles of 1.25% *w*/*v* DSS during the 63-day period was not influenced by consumption of an iron-deficient diet (100 ppm iron [blue]), standard chow diet (200 ppm iron [red]) nor an iron supplemented diet (400 ppm iron [green]); Data presented as mean ± standard error of the mean (SEM). No statistical differences were seen between groups (*n* = 8 mice per group). (**b**) Percentage body weight loss in mice resulting from DSS-induced acute colitis on 63 days on deficient and supplemented iron diets compared with standard chow (*n* = 4 female mice per group). Statistical differences * *p* < 0.05, ** *p* < 0.01, *** *p* < 0.001; Kruskal–Wallis test followed by multiple comparison tests.

Chronic colitis was induced in C57BL/6 mice by three treatment cycles of 1.25% *w/v* DSS in their drinking water. At autopsy, all mice showed histological evidence of mild chronic colitis. Chronic colitis-induced weight loss in C57BL/6 mice was not influenced by altering dietary iron: all mice, irrespective of the iron content of their diet, lost significant body weight from day 6, with maximal weight loss occurring at day 8 of each of the DSS treatment cycles (Figure 1a). Although mice receiving an iron-deficient diet (100 ppm iron) appeared to lose more weight than other groups, this difference was not significant (Figure 1a). Non-DSS treated control mice, irrespective of dietary iron content, all showed steady increases in body weight. However, mice fed a 400 ppm iron supplemented diet showed significantly greater weight gain throughout the 63-day study; all sampling points *p* < 0.05; *n* = 4, Kruskal–Wallis test (Supplementary Information, Figure S2).

### 2.2. Acute Colitis Is More Severe in Mice Fed Long-Term on an Iron Deficient Diet

**Figure 2:**
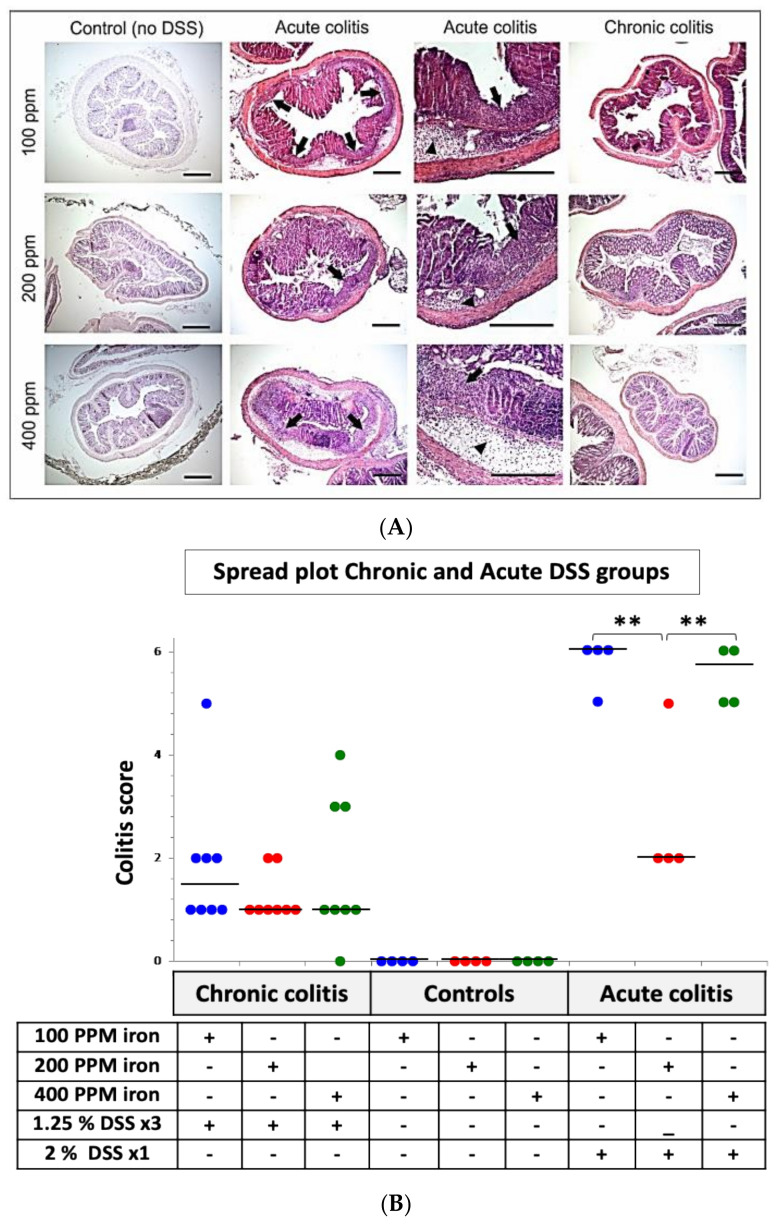
Histological analysis of colon from DSS-treated mice in consuming diets of differing dietary iron levels. (**A**) Representative hematoxylin and eosin-stained segments of distal colon from control C57BL/6J mice ingesting 100, 200 or 400 ppm iron diets alone (*n* = 4 mice in total), C57BL/6 with acute (*n* = 4) and chronic DSS-induced colitis (*n* = 8) administered with 100, 200 or 400 ppm iron diets as indicated. Arrowheads highlight submucosal oedema; arrows highlight almost complete loss of colonic epithelium and leukocyte infiltration. Scale bar: 200 µm. (**B**): Inflammation (colitis) scores for all groups’ DSS-treated (24 (63-days) and 12 (10-days) mice per group) and untreated (controls) 12 (63-days) mice on different iron diets. Horizontal lines at the median. an iron deficient diet (100 ppm iron [blue]), standard chow diet (200 ppm iron [red]) nor an iron supplemented diet (400 ppm iron [green]) Differences tested by Kruskal–Wallis test followed by multiple comparison tests ** *p* < 0.01.

Following 53-days consuming one of three diets of differing iron content, mice ingesting 2% *w/v* DSS for 5-days in their drinking water developed moderately severe acute colitis. Mice ingesting 2% *w/v* DSS for 5-days in their drinking water also lost significant body weight with acute colitis. Weight loss began earlier (day 4) in those ingesting an iron-deficient (100 ppm) diet compared to mice consuming standard (200 ppm) and iron-supplemented (400 ppm) chow (*p* < 0.001; *n* = 4; Kruskal–Wallis test (Figure 1b). Indeed, mice fed the iron-deficient diet lost significantly more weight (peak weight loss at day 8, *p* < 0.001; *n* = 4, Kruskal–Wallis test) than the other treated groups from day 4 to day 8. The colons of control mice, that had not received DSS, appeared histologically normal (see Figure 2A). The colitis scores were significantly greater in mice that had been treated with 2% *w/v* DSS after consuming either the iron deficient (100 ppm) diet or the iron supplemented (400 ppm) diet, compared to mice on standard chow (*p* < 0.01; *n* = 4, Kruskal–Wallis test) and compared to mice that received three cycles of 1.25% *w/v* DSS (Figure 2B).

### 2.3. Intestinal Fibrosis Is Increased in Mice with Chronic Colitis Fed an Iron Deficient Diet

Masson’s trichrome staining was used to assess the level of fibrosis in the colons of mice with DSS-induced chronic colitis. As expected, the colon of all mice with chronic colitis showed increased levels of fibrosis, as evidenced by increased presence of collagen and proteoglycan within the mucosa and submucosa) compared to non-DSS treated animals (*p* < 0.01, *n* = 8 mice per group; Kruskal–Wallis test). Mice with chronic colitis fed the iron-deficient diet (100 ppm iron) had markedly more fibrosis evident (*p* < 0.05, *n* = 8; Kruskal–Wallis test) than those the mice treated with DSS consuming standard and iron supplemented diets (see Supplementary Information—Figure S3).

### 2.4. Fecal Calprotectin Changes in Colitic Mice Fed Differing Iron Diets

A trend of increasing level of calprotectin detected in feces was seen following each cycle of 1.25% *w/v* DSS treatment in mice consuming altered (deficient and supplemented) iron diets for 63-days compared to those mice consuming standard chow. However, significant differences were only seen in colitic mice consuming the high iron supplemented (400 ppm) chow at day-21 and day-63; both *p* < 0.01, *n* = 8 per group; Kruskal–Wallis test (Supplementary Information—Figure S4). Following 53-days consuming one of three diets of differing iron levels, induction of moderately severe acute colitis over the following 10-days led to significantly increased calprotectin in the feces. The change in fecal calprotectin appears to be greater in mice with acute colitis consuming the 100 ppm iron-deficient diet than seen in the other experimental groups (Supplementary Information—Figure S4). No changes were seen in non-DSS-treated mice on the three respective diets.

### 2.5. Fecal Iron Changes in Colitic Mice Fed Differing Iron Diets

**Figure 3:**
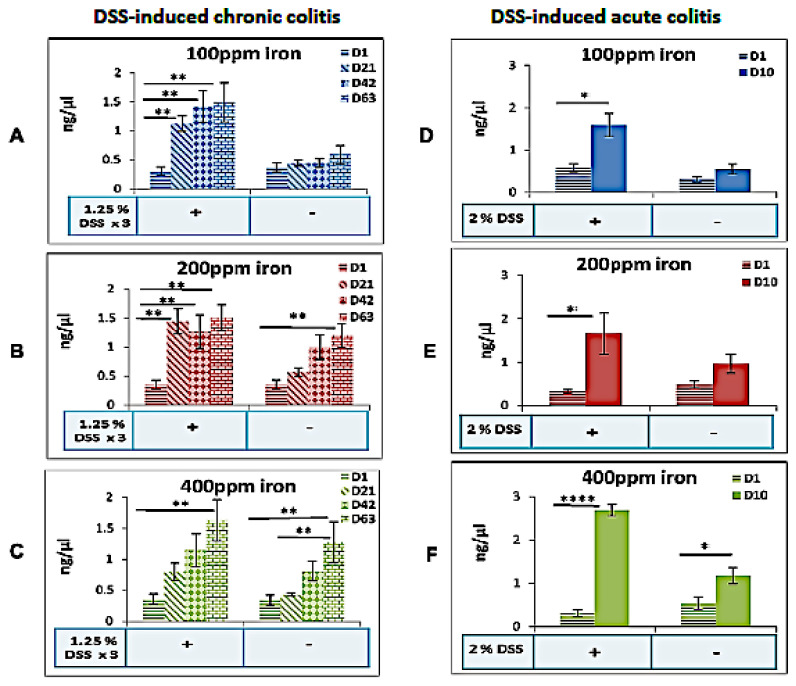
Fecal iron changes in mice fed differing iron diets in the presence or absence of colitis. Fecal iron concentrations in the presence or absence of DSS-induced chronic DSS (day-1 vs. day-21, day-42, and day-63; *n* = 8 mice per group) or acute colitis DSS (day-1 vs. day-10; *n* = 4 mice per group), for mice consuming a (**A**,**D**) deficient (100 ppm iron), (**B**,**E**) standard (200 ppm iron) or (**C**,**F**) supplemented (400 ppm iron) chow diet respectively. Data are presented as a mean ± standard error of the mean (SEM). Differences were tested by Kruskal–Wallis test followed by multiple comparison Dunn’s test; * *p* < 0.05, ** *p* < 0.01, **** *p* < 0.0001.

In the chronic colitis study, DSS-treated mice consuming a high (400 ppm) supplemented iron diet showed an increase in fecal iron levels at day-63 compared to day-1 (2.6-fold increase). Groups ingesting the iron deficient (100 ppm) and standard iron (200 ppm) diets, and treated with DSS to induce colitis, both showed significant increase in fecal iron levels at day-21, day-42, and day-63 compared to levels measured on day-1 (Figure 3), consistent perhaps with the presence of luminal iron from bleeding resulting from colitis. In control mice, fecal iron was only seen to be significantly increased at day-63 when ingesting standard (200 ppm) and supplemented (400 ppm) diets, and not in mice consuming the low (100 ppm) iron diet (Figure 3).

Following induction of acute colitis, fecal iron concentration increased significantly in all dietary study groups; all *p* < 0.05 (Figure 3). This was more pronounced in mice ingesting higher levels of iron, i.e., the iron supplemented (400 ppm) diet.

### 2.6. Bacterial Diversity in Mice Fed Differing Levels of Dietary Iron and Following Induction of Chronic Colitis

Tables of rarefied OTU data were prepared, and three measures of alpha diversity were estimated: chao1, the observed number of species, and the phylogenetic distance. These estimates were plotted as rarefaction curves using Qiime (see Supplementary Information—Figure S5). Weighted and unweighted pairwise UniFrac matrices UPGMA trees were prepared for measure of beta-diversity (Supplementary Information—Figure S6).

**Figure 4:**
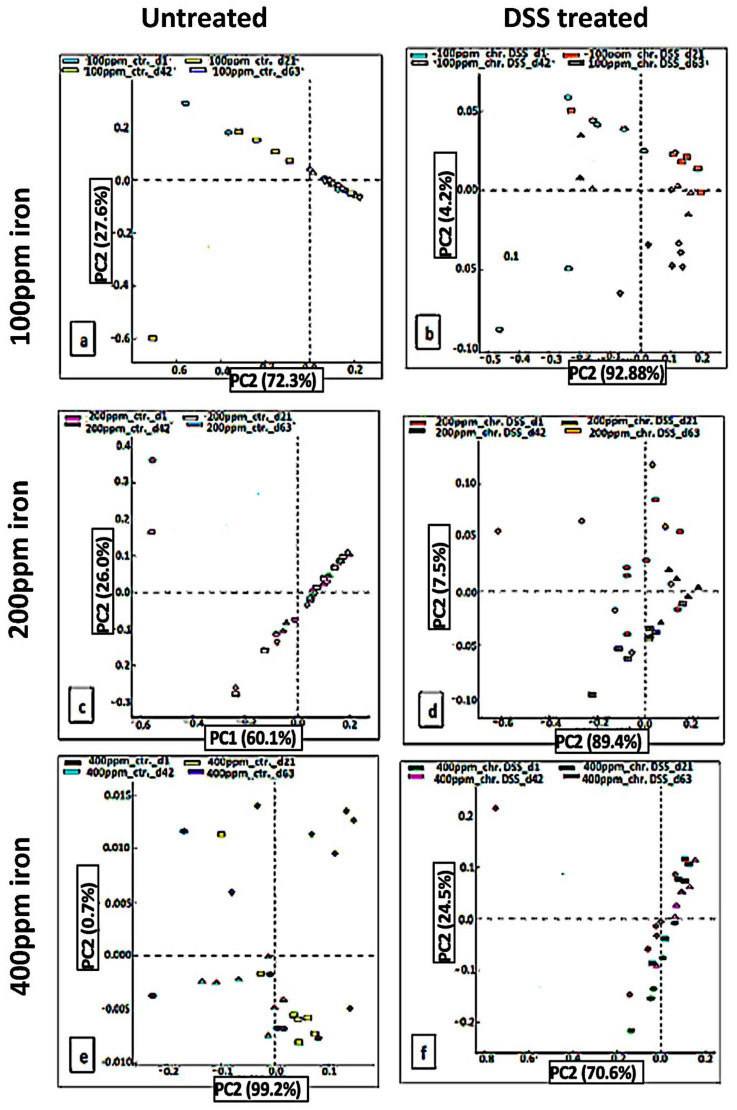
PCA to show unweighted UniFrac diseases after DSS treatment. In chronic DSS, PCA plots of the unweighted UniFrac distances of pre-and post-DSS-intervention stool samples from chronic (3 cycles) DSS – treated mice (**b**,**d**,**f**) and (**a**,**c**,**e**) untreated mice at phylum-level, phylogenetic classification of 16S rRNA gene sequences. Symbols represent data from individual mice, color-coded by the indicated metadata. Statistical differences were assessed by Kruskal–Wallis H-test followed by Storey’s FDR multiple test correction.

Principal component analysis (PCA) identified linear combinations of fecal microbial taxa associated with the duration of the test diets (Figure 4). Data showed overlap in identified taxa within fecal samples obtained from mice fed the low iron (100 ppm) diet, mice on the standard iron (200 ppm) diet, and in mice with chronic colitis induced by DSS ingesting the standard iron diet, from days 1, 21, and 42 to day-63 (Figure 4a,c,d). However, there was clustering with little separation of samples pre- and post-DSS treatment who were fed the low iron (100 ppm) diet and the high (400 ppm) iron diet, as well as mice fed the high iron (400 ppm) diet alone (Figure 4b,e,f). The high iron (400 ppm) diet altered the microbial community significantly in both DSS-treated and untreated mice.

**Figure 5:**
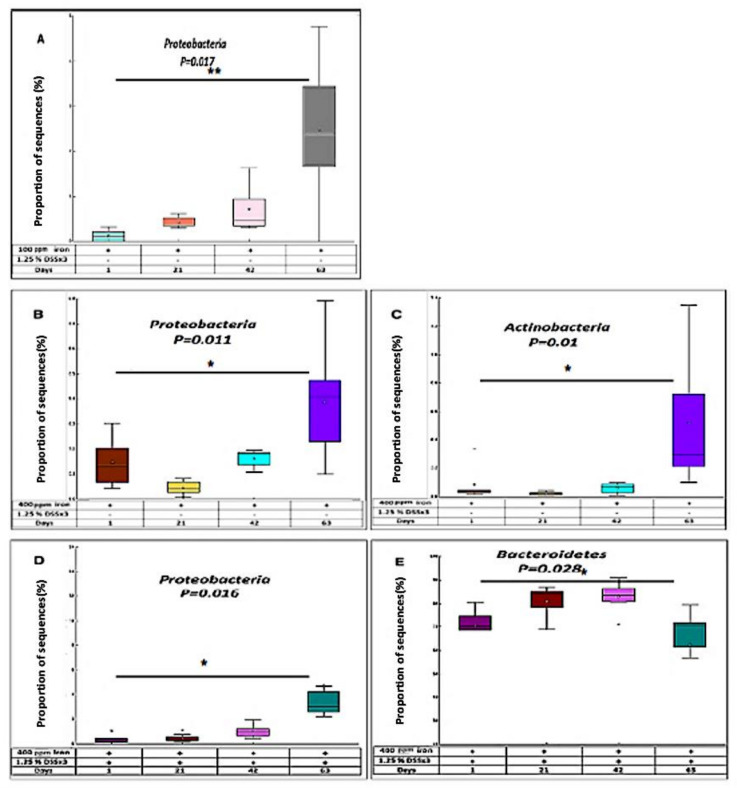
Distribution of bacteria in response to chronic DSS. (**A**) In chronic DSS, box plot showing the distribution in the proportion of *Proteobacteria* assigned to samples at day-1, 21, 42, and 63 from 100 ppm iron DSS-treated mice. In chronic DSS, box plot showing the distribution in the proportion of two phyla (*Proteobacteria* (**B**) and *Actinobacteria* (**C**)) assigned to samples from 400 ppm iron untreated mice. In chronic DSS, box plot showing the distribution in the proportion of two phyla (*Proteobacteria* (**D**) and *Bacteroidetes* (**E**)) assigned to samples from 400 ppm iron DSS-treated mice.

Post-hoc tests revealed a significant difference in the amount of *Proteobacteria* in chronic DSS-treated mice ingesting a low (100 ppm) iron diet when day-1 and day-63 were compared (*p* < 0.017, Kruskal–Wallis H-test) (Figure 5A). In control mice fed the high (400 ppm) iron diet, there was a significant increase in two key phyla, *Proteobacteria* and *Actinobacteria*, when comparing day-1 and day-63 samples (*p* < 0.05 for both) (Figure 5B,C). The analysis of fecal samples from mice treated with DSS fed the high (400 ppm) iron diet showed alterations in abundance of *Bacteroidetes* and *Proteobacteria* comparing day-1 and day-63; with a significant decrease observed in *Bacteroidetes* (*p* < 0.05) and increase in *Proteobacteria* (*p* < 0.05) (Figure 5D,E).

Using STAMP software encourages the use of effect sizes and confidence intervals to identify significance [27]. Further bioinformatics analysis identified 4 phyla (*Firmicutes, Bacteroidetes*, *Proteobacteria,* and *Actinobacteria*) and 15 taxa of interest. The results of the relative abundances of various phyla and identified genera were found only for iron-deficient diet, DSS-treated group, and for high iron diet groups (both DSS-treated and untreated) are summarized in Table 1a–c. Of the four phyla identified, *Firmicutes* were highly abundant within samples from all dietary and treatment groups, whilst *Actinobacteria* were seen to be less abundant. In the DSS-treated mice fed the iron deficient (100 ppm) diet, we found marked reduction in six genera, including *Lactobacillus* (corrected *p =* 0.002), *Dorea*, *Clostridium*, *Bacteroides*, *Odoribacter* (all *p* < 0.05) and *Bilophila* (*p =* 0.008), and an increase in *Prevotella* (*p =* 0.008). In the DSS-treated group fed the high (400 ppm) iron diet, a significant reduction was observed in the relative abundance of *Lactobacillus* (*p =* 0.01). The only control group where significant differences in the relative abundance of taxa were found was the supplemented (high) 400 ppm iron group, where with the seven genera showed a statistically significant difference. Increases were shown in *Lactobacillus* (corrected *p =* 0.02), *Oscillospira* (*p =* 0.03), *Adlercreutzia* and *Candidatus Arthromitus* (both *p =* 0.04), whereas reductions were observed for *Bacteroides* (*p =* 0.02), *Bilophila* (*p =* 0.03) and *Ruminococcus* (*p =* 0.04) Table 1a–c.

## 3. Discussion

Induction of colitis in mice using DSS is a popular surrogate model for the study of human ulcerative colitis [28]. The mechanism of action of DSS is not well defined and likely a combination of actions, including direct toxic activity on the colonic epithelium [29], induced mucosal infiltration of macrophages [30] and/or alteration of the intestinal microbiota [31,32]. More common has been the use of DSS to induce an acute colitis; however, Okayasu and colleagues have described a chronic colitis model in mice, which may more closely mirror chronic IBD in humans [28]. We recently reported effects for short-term dietary iron modification in the DSS acute colitis model, using iron deficient (100 ppm) and iron supplemented (400 ppm) diets compared to standard chow (200 ppm dietary iron), where both deficient and supplemented iron diets were associated with more severe colitis than seen with a standard chow diet [19]. Here, we report the effects of the same dietary modifications in a model of DSS-induced chronic colitis, and also the effects of long-term prior dietary modification on a model of acute colitis, so as to mimic relapse of IBD following iron supplementation or deficiency in patients.

We found that when acute colitis was induced after 7 weeks of dietary iron modification, mice consuming the iron-deficient (100 ppm) or iron supplemented (400 ppm) diet also developed more severe colitis than mice ingesting standard chow. Both clinical and histological data were concordant for the iron deficient (100 ppm) group. Our previous work had shown that reduced dietary iron intake was associated with increased weight loss and more severe colitis following the induction of acute colitis using DSS [19]. In contrast, mice in which chronic colitis was induced using DSS, whether consuming dietary iron levels of 100 ppm, 200 ppm, or 400 ppm, showed only modest weight loss and histological colitis. We must acknowledge that the differences in weight may be a reflection of iron deficiency per se, and not entirely due to colitis.

Fecal calprotectin changes were seen in our colitic mice fed differing iron diets, being greater in those animals consuming an iron-deficient diet in the acute colitis model, and with the iron supplemented diet in the chronic colitis model, most likely reflecting an enhanced protective response against inflammation-induced damage [33].

As expected, supplementation of dietary iron led to an increase in fecal iron levels and following induction of chronic colitis, fecal iron was increased in all mice irrespective of levels of dietary iron intake. In the acute DSS experiment, mice ingesting the iron supplemented (400 ppm) diet showed the most marked increases in fecal iron levels. There is an obvious paradox: reducing dietary iron was associated with an increase in loss of iron in feces. The mechanism appears to be exacerbating DSS-induced colitis. We speculate that the iron-deficient diet led to more severe colitis, which secondarily led to an increase in bleeding and hence the observed increased in fecal iron levels. Therefore, iron deficiency and DSS-induced colitis pathogenetic mechanisms are synergistic [27]. However, it is necessary to further explore the specific mechanism.

Changing dietary iron concentrations led to a significant alteration in the intestinal microbiota, both in mice with chronic colitis fed the iron-deficient (100 ppm) and the iron supplemented (400 ppm) diet, and in control (non-colitic) mice ingesting the high iron (400 ppm) diet. Previous research has established a reduction in the biodiversity of commensal bacteria in IBD [34,35,36,37]. In mouse experiments, changes in bacterial composition have been observed as a consequence of colonic inflammation and infection [17,38], with particular intestinal pathogens (species from within the phylum *Proteobacteria*) appearing to take advantage of this inflammatory environment. This observation is in agreement with the ‘food hypothesis’ and ‘differential killing’ hypothesis [39]. These two mechanisms are likely to contribute to the loss of colonization resistance in the inflamed bowel [39]. The post-hoc analysis of our data revealed that *Proteobacteria* were increased significantly, particularly in fecal samples obtained from mice with chronic colitis fed an iron deficient (100 ppm) or iron-supplemented (400 ppm) diet or fed the latter diet in the absence of inflammation. In other words, there was a significant association between fecal iron levels and abundance of *Proteobacteria*. Meanwhile, a relative abundance of *Bacteroidetes* was observed to be lower in colitic mice fed on the high iron (400 ppm) diet. Together these data suggest that *Proteobacteria* are dependent on luminal iron, but *Bacteroidetes* are suppressed by inflammation and/or luminal iron. However, acute DSS groups post-hoc analysis did not reveal any significance this could be, due to either low number of mice (4 per each group) or the detergent effect of 2% DSS on microbiota which caused desctuction to those microorganisms. 

Investigation of the effects of dietary iron upon the murine gut microbiota has been previously described by the Haller group [40], where they identified eight bacterial families and nine genera were significantly affected by luminal iron deficiency: *Bifidobacterium* (*p* < 0.0018), *Succinivibrio*, *Turicibacter,* and *Clostridium* genera were significantly increased in mice fed an iron-depleted diet, whereas the genera *Desulfovibrio*, *Dorea,* and *Bacteroides* were greatly reduced [41]. Our data analysis showed that the relative abundance of seven genera were significantly altered with respect to dietary iron and colitis. Mice with chronic colitis-induced by DSS and fed either low (100 ppm) or high (400 ppm) iron diet, showed marked differences in seven genera belonging to three phyla *Firmicutes*, *Bacteroidetes,* and *Proteobacteria* within the fecal microbiota. Reduction in *Lactobacillus* was seen with both diets, whilst reductions in genera *Dorea*, *Clostridium*, *Bacteroides*, *Odoribacter,* and *Bilophila* were observed only with the iron-deficient diet, along with a significant increase in *Prevotella*. Impact on the fecal microbiota of dietary iron alone was only seen with mice fed the high iron (400 ppm) diet, with seven genera (from four phyla *Firmicutes, Bacteroidetes, Proteobacteria,* and *Actinobacteria*) showing marked change, including increase in *Lactobacillus*, *Oscillospira*, *Adlercreutzia,* and *Candidatus Arthromitus*, and reduction observed for *Bacteroides*, *Bilophila,* and *Ruminococcus.* Haller et al. concluded that all significant differences in bacterial abundance in wild-type mice likely appeared as a result of the interaction between treatment and host-mediated inflammation [40,42]. There are several key differences between that paper and our own: they investigated cecal contents, not feces; they induced ileitis, not colitis and they did not measure fecal iron concentration. Studies conducted by Sartor et al. highlight that changes in dietary iron intake likely modify the biological structure of the intestinal microbiota and level of inflammation [43], and agree well with our work presented here.

In the presence of colitis, dietary iron at 400 ppm resulted in a significant reduction in fecal abundance of *Firmicutes* and *Bacteroidetes*, and increase of *Proteobacteria*, changes which were not observed with lower dietary intake of iron at 100 ppm. Overall, altering dietary iron intake exacerbated DSS-induced colitis; increasing the iron content of the diet also led to changes in intestinal bacteria diversity and composition after colitis was induced with DSS. The successful and reproducible induction of DSS-induced colitis was achieved in our previous work [19] allowed us to obtain fecal samples to assess microbiota disturbances at various time-points. Therefore, we were encouraged to observe long-term changes in nutritional luminal iron and their effect on colitis as well as dysbiosis in mice with acute or chronic colitis.

The multi-factorial mechanisms by which IBD-associated dysbiosis develops are not fully understood, and it is unclear whether this dysbiosis should be considered a cause or consequence of IBD. Luminal iron likely to contributes to the dysbiosis associated with IBD [44,45,46]. In a recent preliminary study, our group has shown that when luminal iron increased a dysbiosis occurs especially in IBD patients who have IDA (iron deficiency anemia) as well as in active IBD [47]. *Proteobacteria* was obviously an iron-responsive microbiota as presence of iron in the gut promotes their growth and apparently contribute to the excess of this phylum during relapse. However, in the same study, *Bacteroidetes* seemed to be independent of luminal iron, unlike *Firmicutes*. Though, a further investigation required clarification of the exact effect of lack as well as excess of iron upon the colonic microbiome [47].

Obtaining iron is an important factor for bacterial pathogenicity, and recent discoveries have connected this to the expression of virulence factors in normal gut microbiota that have become pathobionts [48]. Though, dietary iron clearly plays a role in modulating the susceptibility to DSS-induced colitis as described here, and in our previous study [27]. As it has been shown in our data, a lower amount of iron in the diet has exacerbated the gut microbiota as well as excess iron diet. This was partly agreed with other previous studies that showed iron fortification in children’s diet has a potential production of further pathogenic gut microbiota profile occurred, and this was linked with increased gut inflammation [42].

The data presented are based on rodents with a single model of colitis and we did not study humans. However, the impact on the microbiome is determined by the microbial community and it is plausible that similar changes in the intestinal bacteria will be seen in humans to those we report in mice. We also acknowledge that a single form of iron supplementation was used: it is likely that other forms of ferrous iron supplementation will have similar effects, but we cannot extrapolate the observation to newer ferric formulations.

## 4. Materials and Methods

### 4.1. Animals

Female C57BL/6 mice, aged between 8 to 9 weeks old, were purchased from Charles River Laboratories (Margate, UK). Mice initially received standard chow diet and water *ad libitum*, during an acclimatization period of at least one week. Animals were then individually caged in a room with controlled temperature, humidity, and a pre-set dark: light cycle (12 h:12 h) in a specific pathogen-free animal facility. For experiments, mice were matched for age and body weight. Six groups of 8 mice were studied: 3 control groups and 3 groups where colitis was chemically-induced, with each group receiving one of 3 diets of differing iron levels. All were maintained for up to 63-days. The care of, and experimentation on, mice was carried out in accordance with UK Home Office regulations (project license no: 70/8457) and the project was reviewed by the University of Liverpool Animal Welfare and Ethical Review Body (AWERB)**.**

### 4.2. Diets

When eating a normal (standard) chow diet, mice were fed CRM (P) Rat and Mouse Breeder and Grower 10 mm compression pellets (Special Diets Services; Witham, Essex, UK) which contain 200 parts per million (ppm) iron (ferrous sulphate), i.e., 0.02% *w*/*w* iron. Two modifications applied to the standard chow: First, reducing the content of iron by 50% result in CRM (P) iron-deficient diet containing only 100 ppm iron, second, doubling the content of iron in diet giving us a CRM (P) iron supplemented diet of 400 ppm iron.

### 4.3. Induction of Colitis

Three groups of 8 mice, consuming deficient, standard, and supplemented iron diets respectively, were given a 1.25% *w/v* dextran sulfate sodium (DSS) (M.W. 36,000–50,000 Da; Catalogue number: 160110; Lot number: 6683K; MP Biomedicals, LLC., UK) in their drinking water for 5-days to induce colitis. Mice were allowed to recover for 16 days and then treatment was repeated, with a total of three cycles of DSS used to induce a chronic colitis that models chronic IBD [49], and which is associated with the development of fibrosis [50].

Eight mice in three control groups also received the standard, iron deficient or supplemented diets respectively for 63-days. After 53-days, each group was divided into two, half (*n* = 4) carried on as controls and the other half were treated with 2% *w/v* DSS for 5-days in drinking water, followed by 5-days of plain drinking water, to induce an acute colitis. All mice were euthanized on day-63. A schematic of treatments and diets can be found in Supplementary Information, Figure S1.

### 4.4. Histopathological Scoring of Colonic Inflammation

The distal colon was removed, fixed in 4% *v/v* neutral-buffered formalin, dehydrated, wax-embedded, and then 4 μm sections were cut by microtome. Sections were stained with hematoxylin and eosin (H and E) and evidence of colitis was reported using the inflammatory scoring system described by Bauer et al. [51]. Fibrosis (collagen and proteoglycan deposition) was evaluated using a NovaUltra^TM^ Masson’s Trichrome Stain Kit (Fisher Scientific UK Ltd., Loughborough, UK) [52]. The experimenter was blinded to the treatment groups while all slides were assessed.

### 4.5. Assessment of Degree of Gut Inflammation through Measurement of Faecal Calprotectin

Feces was collected from each mouse, on day-1, 21, 42, and 63 in the chronic colitis study, and on day-1 and day-10 of the acute colitis study, and from control mice. Fecal calprotectin concentration was prepared and measured as per manufacturer instructions using a S100A8/S100A9 ELISA kit (Immundiagnostik AG; Bensheim, Germany).

### 4.6. Measurement of Fecal Iron

Using the same fecal pellets that were collected for calprotectin assessment, the level of iron (Fe^2+^ and Fe^3+^) was measured using an iron immunoassay kit (MAK025; Sigma-Aldrich Company Ltd., Gillingham, UK).

### 4.7. Fecal Bacterial DNA Extraction and Sequencing

Fecal samples (2 g) were also collected and bacterial DNA isolated using the PSP^®^ Spin Stool DNA Plus Kit (STRATEC Molecular GmbH, Berlin, Germany) following the manufacturer protocol. Isolated DNA was provided to the Centre for Genomic Research (University of Liverpool, Liverpool, UK) to generate the 16S Metagenomic Sequencing Library using primers described by Caporaso et al. [41] to amplify the V4 region of 16S rDNA:

F: 5’CACTCTTTCCCTACACGACGCTCTTCCGATCTNNNNNGTGCCAGCMGCC GCGGTAA3′

and R: 5’GTGACTGGAGTTCAGACGTGTGCTCTTCCGATCTGGACTACHVGGGTW TCTAAT3’.

Five µg of DNA was used for first-round PCR with conditions of 20 s at 95 °C, 15 s at 65 °C, and 30 s at 70 °C for 10 cycles, then a final 5-min extension at 72 °C. Samples were purified using Axygen SPRI Beads. The second-round PCR was performed to incorporate Illumina sequencing adapter sequences: 15 cycles of PCR were performed using the same conditions. Samples were re-purified then quantified using Qubit and assessed using the fragment analyser. Successfully-generated amplicon libraries were sequenced [19].

The final libraries were pooled in equimolar amounts using the Qubit and fragment analyser data and 350--550 bp size-selected on the Pippin Prep. The quantity and quality of each pool were assessed by Bioanalyzer and subsequently by qPCR using the Illumina Library Quantification Kit from Kapa on a Roche Light Cycler LC480II according to manufacturer’s instructions. The pool of libraries was sequenced on one lane of the MiSeq at 2 × 250 bp paired-end sequencing. To help balance the complexity of the amplicon library 15%, PhiX was spiked in [19].

### 4.8. Bioinformatics

Initial processing and quality assessment of the sequence data was performed using an in-house pipeline. Base-calling and de-multiplexing of indexed reads were conducted by CASAVA version 1.8.2 (Illumina). The raw fastq files were trimmed to remove Illumina adapter sequences where any reads that matched the adapter sequence over at least three bp were trimmed off. The reads were further trimmed to remove low-quality bases (reads <10 bp were removed). Read pairs were aligned to produce a single sequence for each read pair that would entirely span the amplicon. Sequences with lengths outside the expected range were excluded [19]. The sequences passing the above filters for each sample were pooled into a single file. A metadata file was created to describe each sample. These two files were analyzed using Qiime, version 1.8.0 [53]. Similar sequences were clustered into groups, to define OTUs of 97% similarity. OTU-picking was performed using USEARCH7 [54]. The Greengenes database version 12.8 [55] was used for reference-based chimaera detection [19]. OTU tables were repeatedly sub-sampled (rarefied) and for each rarefied OTU table, three measures of alpha diversity were estimated: chao1, the observed number of species, and the phylogenetic distance. For inter-sample comparisons (beta-diversity), all datasets were rarefied and tables were used to calculate weighted and unweighted pairwise UniFrac matrices using Qiime. UniFrac matrices were then used to generate UPGMA (Unweighted Pair-Group Method with Arithmetic mean) trees and 2D principal coordinates plots [19].

### 4.9. Statistics

Normally distributed physiological and biochemical data were assessed by one-way analysis of variance (ANOVA) followed by selected pair-wise comparisons using Dunn’s test. Non-normally distributed data have been evaluated by Kruskal-Wallis test followed by pairwise multiple comparisons using StatsDirect v3.0.171; StatsDirect Ltd., Birkenhead, UK). For the bioinformatic analysis of microbiota data, Kruskal–Wallis H-test was used with the false discovery rate (FDR) Storey’s (multiple correction tests). The q-value is the adjusted *p*-value based on FDR calculation, where statistical significance was declared at *p* < 0.05.

## 5. Conclusions

Lowering iron content to 100 ppm in rodent diet significantly exacerbated acute colitis leading to an increase in fecal iron, whereas, increasing iron levels to 400 ppm resulted in significant alteration in gut microbiota. Consequently, this explains the important role that luminal iron plays in pathogenesis of inflammation in the gut. Additionally, the alteration in dietary iron over a longer period can significantly exacerbate susceptibility to DSS-induced intestinal inflammation, suggesting that the tenure of iron supplementation may also be crucial in aggravating colitis. Further studies will be necessary to investigate the relevance of our findings in humans.

## Figures and Tables

**Table 1:** (**a**): Genus-level taxonomic composition of fecal samples from 100 ppm iron DSS-treated mice (Day-1 vs. 21, 42, and 63 samples), (**b**) Genus-level taxonomic composition of fecal samples from 400 ppm iron DSS-treated mice (Day-1 vs. 21, 42, and 63 samples), (**c**) Genus-level taxonomic composition of fecal samples from 400 ppm iron untreated mice (Day-1 vs. 21, 42, and 63 samples).

**(a)**			
**100 ppm iron DSS-treated group**			
**Taxon**	***p*-values**	***p*-values (corrected)**	**Effect size**
p_*Bacteroidetes*; g_*Bacteroides*	0.003	0.047	0.496
p_*Bacteroidetes*; g_*Odoribacter*	0.002	0.04	0.620
p_*Bacteroidetes*; g_*Prevotella*	0.0002	0.008	0.669
p_*Firmicutes*; g_*Clostridium*	0.002	0.04	0.431
p_*Firmicutes*; g_*Dorea*	0.003	0.047	0.138
p_*Firmicutes*; g_*Lactobacillus*	0.00002	0.002	0.880
p_*Proteobacteria*; g_*Bilophila*	0.0002	0.008	0.766
**(b)**			
**400 ppm iron DSS-treated group**			
**Taxon**	***p*-values**	***p*-values (corrected)**	**Effect size**
p_*Firmicutes*; g_*Lactobacillus*	0.0001	0.01	0.74
**(c)**			
**400 ppm iron untreated group** **(Controls)**			
**Taxon**	***p*-values**	***p*-values (corrected)**	**Effect size**
p_*Actinobacteria*; g_*Adlercreutzia*	0.002	0.04	0.49
p_*Bacteroidetes*; g_*Bacteroides*	0.0005	0.02	0.68
p_*Firmicutes*; g_*Candidatus Arthromitus*	0.003	0.04	0.54
p_*Firmicutes*; g_*Lactobacillus*	0.0002	0.02	0.77
p_*Firmicutes*; g_*Oscillospira*	0.001	0.03	0.61
p_*Firmicutes*; g_*Ruminococcus*	0.002	0.04	0.46
p_*Proteobacteria*; g_*Bilophila*	0.001	0.03	0.55

## Data Availability

Data mainly presented and the rest stored at the University of Liverpool laboratories.

## Supplementary Materials

The following are available online at https://www.mdpi.com/article/10.3390/ijms22073646/s1, Figure S1: Schematic illustration of chronic DSS and control groups and the later subdivision at day 53 to start acute DSS colitis experiment, Figure S2: Percentage change in body weight in female C57BL/6 mice consuming diets differing in levels of iron over 63 day, Figure S3: Masson’s trichrome staining of the colonic tissues, Figure S4: Faecal calprotectin concentrations in the presence or absence of DSS-induced colitis, Figure S5: Rarefaction curves of the observed number of species metric for all samples (>500 reads), Figure S6: UPGMA (Unweighted Pair-Group Method with Arithmetic mean) trees.
