# Supplementary material for: Long-Term Iron Deficiency and Dietary Iron Excess Exacerbate Acute Dextran Sodium Sulphate-Induced Colitis and Are Associated with Significant Dysbiosis
Source: Int J Mol Sci. 2021 Mar 31;22(7):3646. doi: 10.3390/ijms22073646 (PMC8037348; doi:10.3390/ijms22073646)

Long-term iron deficiency and dietary iron excess exacerbate acute dextran sodium sulphate-induced colitis and are associated with significant dysbiosis

Mahalhal A, Burkitt MD, Duckworth CA, Hold GL, Campbell BJ, Pritchard DM and Probert CS

Supplementary Information

Figure S1: Schematic illustration of chronic DSS and control groups and the later subdivision at day 53 to start acute DSS colitis experiment.

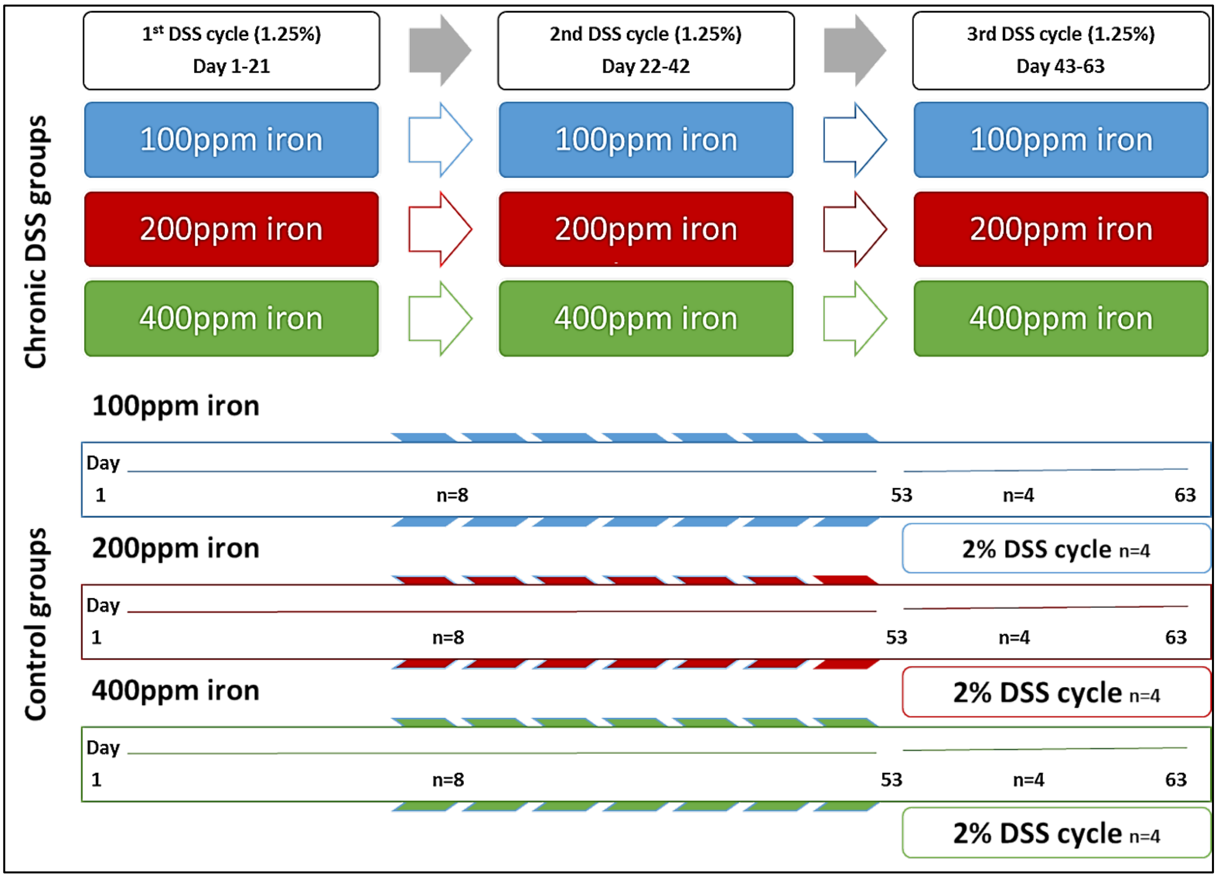

Long-term iron deficiency and dietary iron excess exacerbate acute dextran sodium sulphate-induced colitis and are associated with significant dysbiosis

Mahalhal A, Burkitt MD, Duckworth CA, Hold GL, Campbell BJ, Pritchard DM and Probert CS

Figure S2: Percentage change in body weight in female C57BL/6 mice consuming diets differing in levels of iron over 63 day.

Mice were fed CRM (P) Rat and Mouse Breeder and Grower 10mm compression pellets which contained low iron (100 parts per million (ppm) [blue]), standard levels of iron (200ppm [red] and supplemented levels of iron (400ppm [green]) over a 63-day study period. Data are presented as a mean ± standard error of the mean; [n=8 mice per group (up to day 53), then n=4 (from day 53 to day 63)]. Data were assessed by the Kruskal-Wallis test followed by Dunn's multiple comparisons of treatments comparing day (time point) individually. Statistical differences observed $^{*,\triangledown}p<0.05$, $^{***,\triangledown\triangledown\triangledown}p<0.001$, $^{****,\triangledown\triangledown\triangledown\triangledown}p<0.0001$, comparing the 100ppm ($^{\triangledown}$) & 400 ppm (*) iron groups versus standard 200ppm iron group, respectively.

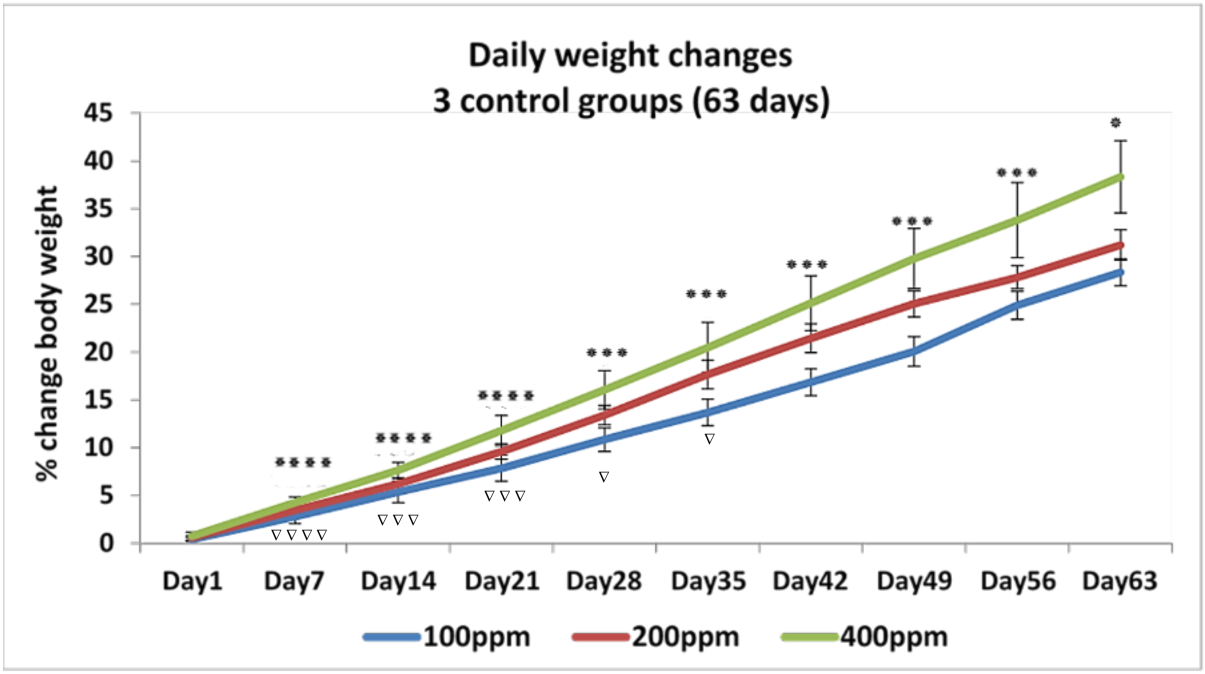

Long-term iron deficiency and dietary iron excess exacerbate acute dextran sodium sulphate-induced colitis and are associated with significant dysbiosis

Mahalhal A, Burkitt MD, Duckworth CA, Hold GL, Campbell BJ, Pritchard DM and Probert CS

Figure S3: Masson's trichrome staining of the colonic tissues of (b) 100ppm iron; (c) 200ppm iron; (d) 400ppm iron mice with dextran sulphate sodium (DSS)-induced colitis at day-63, and 200ppm iron controls (a). Average fibrosis scores for all groups of DSS-treated mice on different iron diets. (e) Horizontal lines at the median. Data are presented as a mean ± standard error of the mean. Differences were tested by Kruskal–Wallis test followed by multiple comparison Dunn's test. (p<0.05).

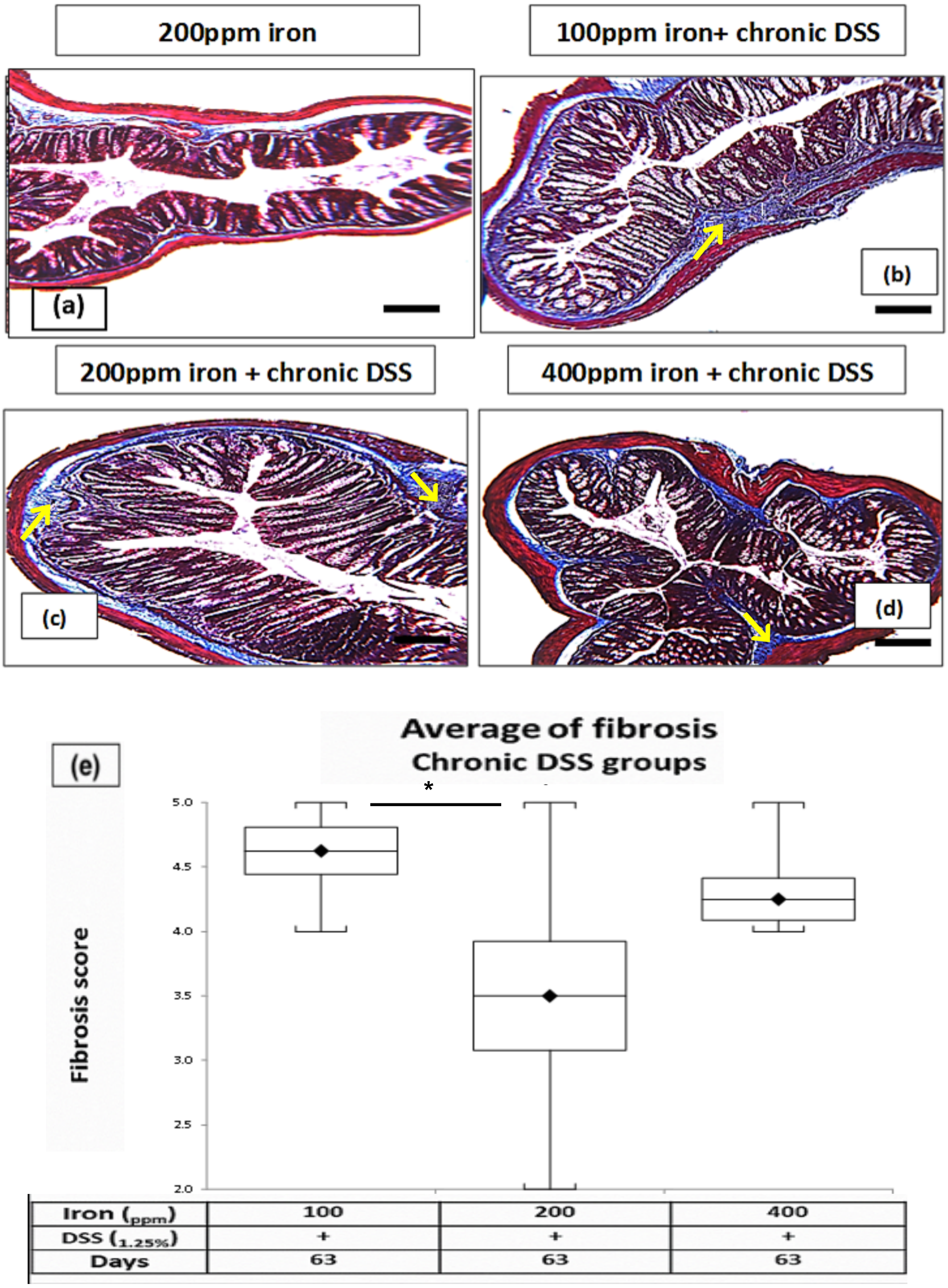

Long-term iron deficiency and dietary iron excess exacerbate acute dextran sodium sulphate-induced colitis and are associated with significant dysbiosis

Mahalhal A, Burkitt MD, Duckworth CA, Hold GL, Campbell BJ, Pritchard DM and Probert CS

Figure S4: Faecal calprotectin concentrations in the presence or absence of DSS-induced colitis **(A)** chronic (day-1 vs. day-21, day-42 and day-63; n=8 for each group) or **(B)** acute colitis (day-1 vs. day-10; n=4 for each group), for mice consuming **(i)** a deficient iron (100ppm iron) diet, **(ii)** standard (200ppm) diet or **(iii)** supplemented (400ppm) iron chow diet. Data are presented as a mean ± standard error of the mean (SEM). Differences were tested by Kruskal-Wallis test followed by multiple comparison Dunn's test; * $p<0.05$ and ** $p<0.01$.

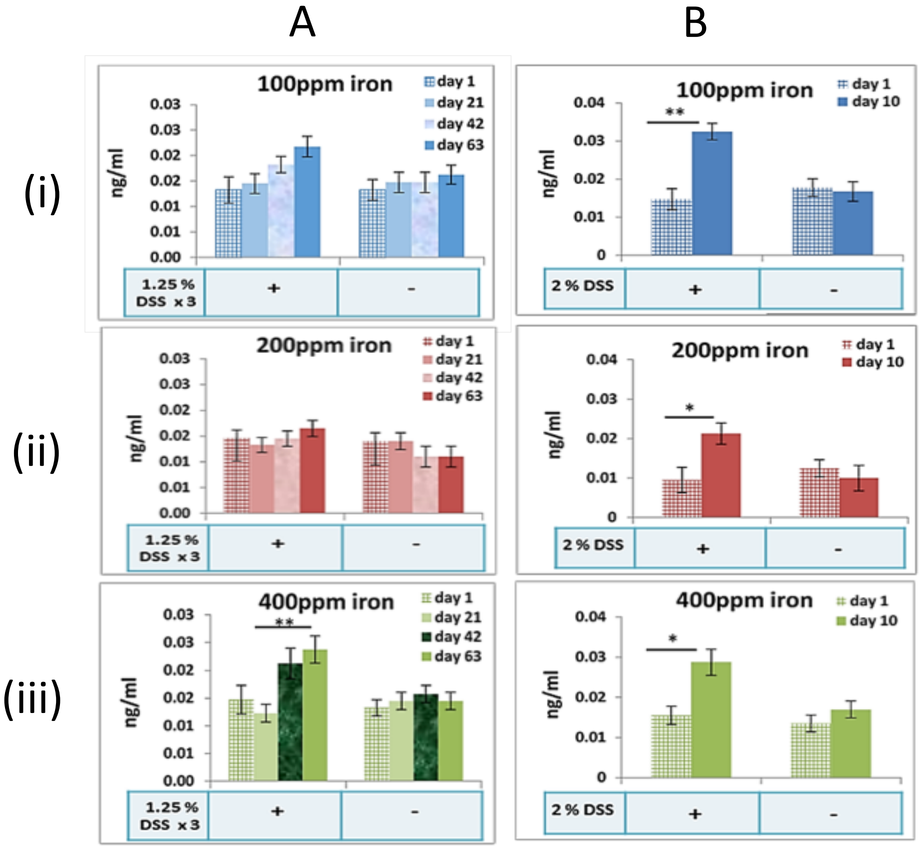

Long-term iron deficiency and dietary iron excess exacerbate acute dextran sodium sulphate-induced colitis and are associated with significant dysbiosis

Mahalhal A, Burkitt MD, Duckworth CA, Hold GL, Campbell BJ, Pritchard DM and Probert CS

Figure S5: Rarefaction curves of the observed number of species metric for all samples (> 500 reads). The plot shows the average number of distinct OTUs found in sub-samples of increasing number of sequences. (A) Chao1 is a nonparametric estimator that predicts the minimum species richness of a sample. (B) The observed number of species is defined as the number of distinct OTUs within a sample. (C) The PD metric represents the minimum total branch length that covers all taxa within the sample on a phylogenetic tree.

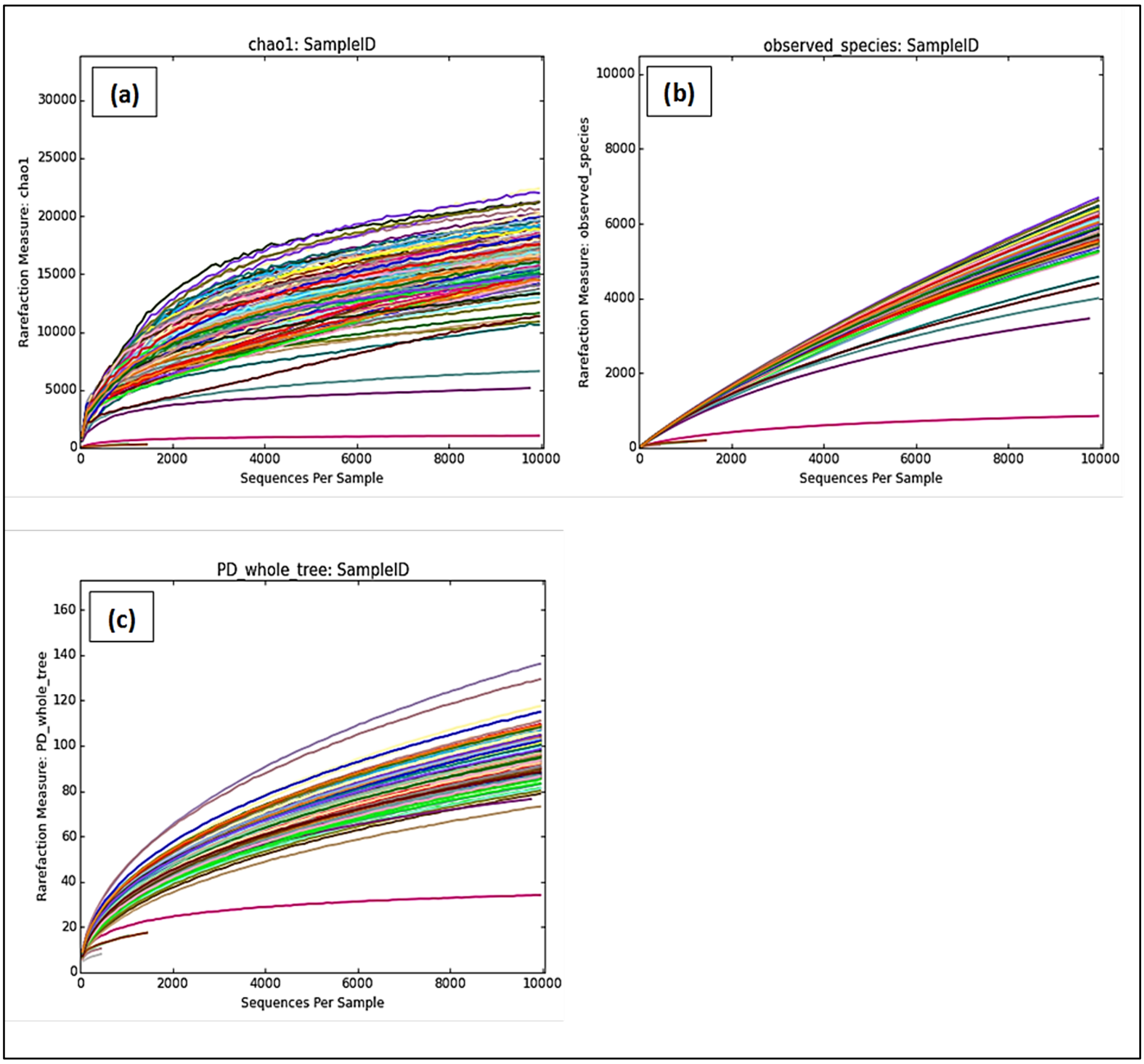

Long-term iron deficiency and dietary iron excess exacerbate acute dextran sodium sulphate-induced colitis and are associated with significant dysbiosis

Mahalhal A, Burkitt MD, Duckworth CA, Hold GL, Campbell BJ, Pritchard DM and Probert CS

Figure S6: UPGMA (Unweighted Pair-Group Method with Arithmetic mean) trees.

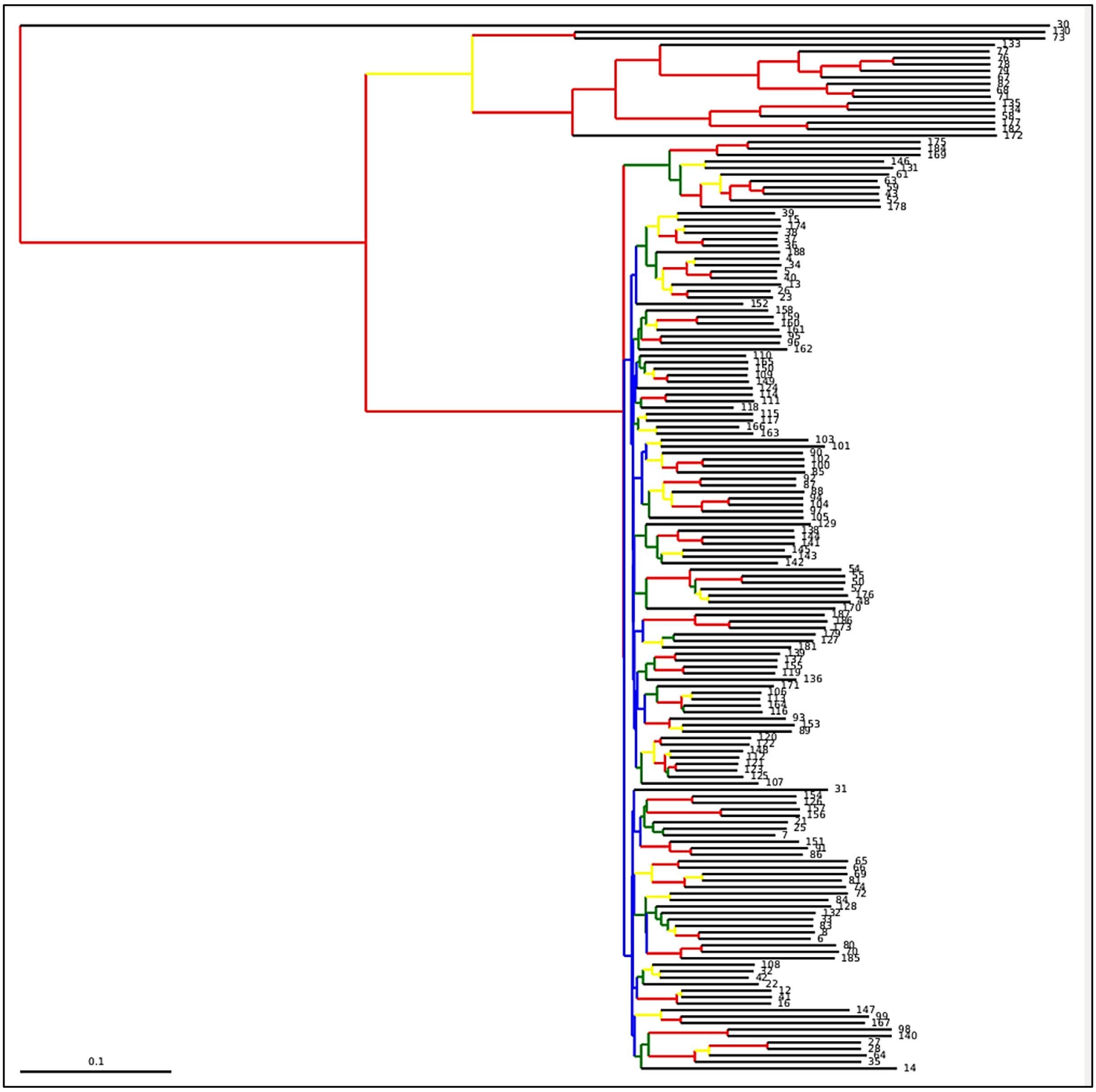

Supplement: Supplementary file 1 [file ijms-22-03646-s001.pdf]
